# High prevalence of *dhfr* and *dhps* molecular markers in *Plasmodium falciparum* in pregnant women of Nchelenge district, Northern Zambia

**DOI:** 10.1186/s12936-015-0676-5

**Published:** 2015-05-06

**Authors:** Mwiche NP Siame, Sungano Mharakurwa, James Chipeta, Philip Thuma, Charles Michelo

**Affiliations:** Department of Public health, University of Zambia, School of Medicine, Lusaka, Zambia; Johns Hopkins Bloomberg School of Public Health, Baltimore, USA; Macha Research Trust, Choma, Zambia

**Keywords:** Zambia, dhfr, dhps, Malaria in pregnancy, SP

## Abstract

**Background:**

Sulphadoxine-pyrimethamine (SP) is the recommended drug for intermittent preventive treatment in pregnancy (IPTp) in most African countries, including Zambia. However, malaria is still one of the leading causes of morbidity and mortality in pregnant women despite reports of greater than 50% of women taking at least two doses of SP in IPTp. Studies have shown that resistance to SP is associated with mutations in the *dhfr* and *dhps* gene of *Plasmodium falciparum*. This study examined the prevalence of *dhfr* and *dhps* polymorphisms in *P. falciparum* found in pregnant women of Nchelenge district.

**Method:**

This cross-sectional study was conducted in 2013 in Nchelenge, a holoendemic area with malaria prevalence estimated at 50% throughout the year. Three rural health centres were randomly selected and a census survey carried out at each health centre. A questionnaire was administered and malaria testing done using RDT and microscopy, with collection of a dried blood spot. A chelex extraction was done to extract parasite DNA from dried blood spots followed by nested PCR and enzyme restriction digestion.

**Results:**

Of the enrolled participants (n = 375), the median age of the women was 23. The prevalence of malaria by PCR was 22%. The PCR positive samples examined (n = 72) showed a high prevalence of *dhfr* triple (Asn-108 + Arg-59 + Ile-59) mutant (68%) and dhps double (Gly -437 + Glu-540) mutant (21%). The quintuple haplotype was found in 17% with 2 samples with an additional Gly-581mutation. In addition 6% mutations at Val-16 were found and none found at Thr-108 respectively, these both confer resistance to cycloguanil. Multivariate analysis showed that there was an association between malaria and women aged 30-34 years old p < 0.05(AOR: 0.36) at 95% CI.

**Conclusion:**

This study showed a high number of mutations in the *dhfr* and *dhps* genes. The high malaria endemicity in the general population of this area may have contributed to the high prevalence of resistant parasites in pregnant women, suggesting a need to examine the efficacy of SP given that it is the only approved drug for IPTp in Zambia.

## Background

Malaria remains a major cause of death predominantly in sub-Saharan Africa. Statistics by the World Health Organization attribute an estimated 627,000 deaths to this disease in 2012 alone [1]. Children under five and pregnant women in high transmission and impoverished areas are at most risk of the disease. Over 30 million pregnant women are estimated to be at risk of *Plasmodium falciparum* malaria in sub-Saharan Africa annually [2]. The pathological effects of malaria adversely affect pregnancy outcome. The unborn child is at increased risk of premature and still-birth, low birth weight, congenital malaria, anaemia and impaired foetal growth [3-5]. Epidemiological settings and host immunity greatly influence the clinical presentation of pregnancy-associated malaria [6-9].

Zambia has achieved over 50% reduction in malaria prevalence in the past decade [10]. However, the challenge remains, as malaria is still one of the leading causes of morbidity and mortality responsible for at least 36% of hospital admissions and 20% of maternal mortality [11]. Sulphadoxine-pyrimethamine is the recommended drug by the World Health Organization for intermittent preventive treatment in pregnancy (IPTp) [12]. IPTp is made available through antenatal care (ANC) at government health facilities and an overall increase in access to ITNs, IRS and IPTp has been reported nationwide [13-15].

In Zambia, IPTp was adopted in 2003. It is administered presumptively three months after quickening (16-26 weeks) [16]. The pregnant woman is given one dose (each containing 500 mg of sulphadoxine and 25 mg pyrimethamine) for every ante natal care visit one month apart, under direct observation of the health worker [17]. Prior to the universal adoption of SP in IPTp randomized controlled trials have showed that SP supplemented with iron decreases the risk of placental parasitaemia, maternal anaemia and low birth weight [18-20].

Drug resistance is one of the greatest challenges of malaria control programmes. Hence, while SP remains ideal for IPTp, in stable transmission areas, increasing resistance has been reported [21,22]. SP resistance is linked to point mutations in the parasite genome specifically the dihydrofolate reductase (*dhfr*) and dihydpteroate synthetase (*dhps*) genes. Mutations in *dhfr* confer resistance to pyrimethamine while mutations in *dhps* confer resistance to sulphadoxine and other sulpha drugs. There are variations in SP mutations, single, double or triple - the more mutations, the stronger the resistance. The *dhfr* triple mutant (Asn-108 + Ile-51 + Arg-59) and *dhps* double mutant (Gly-437 + Glu-540) have been strongly associated with potential resistance in sub-Saharan Africa [23,24].

A study done in 2006 in six districts in the general population of Zambia showed variation of rates of resistance to SP with mutated *dhfr* frequency ranging from 71-92% and 39-71% frequency for the double mutant *dhps,* respectively [25]. Therefore, there is a need for close monitoring and surveillance of the efficacy of SP in pregnant women. This study aimed to investigate the presence of malaria in pregnancy, associated risk factors and determine the prevalence of molecular markers that confer resistance to SP in a highly endemic area.

## Methods

### Design and sampling procedures

This cross-sectional study was conducted in Nchelenge district, Luapula province, Zambia in February to April 2013. The population of Nchelenge is estimated at 180,000 and the inhabitants are mostly fishermen and peasant farmers. Nchelenge has one first level referral hospital St Paul’s Mission hospital, ten rural health centres and three health posts. This is a holoendemic area of malaria transmission with an estimated 50% confirmed cases throughout the year, hence transmission is throughout the year. An estimated 80% of recorded deaths are attributed to malaria with the most affected being children under five years of age and pregnant women. The target population of this study was pregnant women who were residents of Nchelenge and attended antenatal care at any of the three health facilities selected for the study (Table 1). Parental consent and child assent was obtained for those below 18 years old. Non-pregnant women, pregnant women not resident in the study sites or those within the catchment area but severely ill, were not eligible to be included in the study.

**Table 1:** **General characteristics of pregnant women of Nchelenge district (n = 375), 2013**

**Characteristic**	**n or %**
Median age	23 years
Median gestational stage	6-9months
Primigravidae	24%
Multigravidae	54%
Married	86%
No SP intake	15%
>1 dose of SP	64%

Multi-stage sampling was employed. The district was conveniently selected while simple random sampling using Microsoft Excel was used to select the three rural health centres (RHCs) out of 14 in the district and these three were the sites chosen for the study. The rural health centres selected were Kabuta, Nchelenge and Kashikishi RHC. Consenting individuals were included in a census survey from the rural health centres. Hence, all pregnant women who came to the centre for antenatal care, regardless of whether for re-visits or first attendance, were eligible to be included in the study.

The prevalence of malaria in pregnant women was assumed to be 50%, which is the prevalence for the general population in Nchelenge. The calculated total sample size was 384 with a power of 80 at 95% CI.

### Data collection

The data collection tool used in this study was a structured questionnaire to document demographic data and risk factors for the presence of malaria. To validate the data collection tool pre-testing of the questionnaire was done in two ways: the tool was pre-tested to ensure clarity of questions in English and then for consistency, precision, clarity of questions and correct translations in Bemba. Necessary adjustments and revisions were made based on the pre-test results.

### Laboratory methods

The eligible pregnant women were screened for malaria and anaemia on the scheduled ante-natal care day. Malaria was defined as a positive laboratory test for *P. falciparum* (RDT and/or smear and/or PCR). Approximately 100 microlitres of venous blood was collected from each participant by a single finger prick. A rapid diagnostic test (RDT) and microscopy was used to test for malaria. The RDT used detects the antigen histidine rich protein II (HRP II) found on the parasite surface, the test kits brand was the Standard Diagnostics Bioline Malaria antigen Pf test (SD-p.f^TM^) manufactured by Standard Diagnostics Inc., South Korea. The test results were ready within 15 minutes. Thick blood smears were collected on microscope slides for later reading by a microscopist.

Dried blood spots (DBS) were also collected on Whatman 3MM filter paper at the same time as the RDT and thick smear. *Plasmodium falciparum* DNA was extracted from the DBS by a chelex protocol and amplified by polymerase chain reaction (PCR) followed by restriction enzyme digestion. Mutations were defined as the detection of polymorphisms in the *dhfr* gene specifically at Asn-108, Arg-59, Ile-59 and dhps Gly-437, Glu-540 and Gly-581 mutation. Mixed infections were those that showed both sensitive and resistant parasite strains while sensitive strains were parasites that showed no polymorphisms in the region of interest after restriction digestion. Quality assurance was done at Tulane University, School of Public Health and Tropical Medicine in the Kumar Laboratory. Positive samples (n = 10) were randomly selected and PCR was done followed by restriction digestion with enzymes BStNI, AluI and BSrI. The results were similar to those initially obtained hence results were reproducible and reliable.

### Data analysis

Analysis was done using Stata version 12.2. Variables were defined, descriptive statistics; means, medians, standard deviations and odds ratios were used to assess the data. The factors examined were age, marital status, gravidity, education level, ITN ownership and usage, malaria knowledge, medical history and SP intake. These were self reported and SP intake was confirmed by medical records. Chi square was used to determine the association between malaria and each of the associated factors. Significance (p-value) was set at 5% with a confidence interval at 95%. Multivariate analysis using logistic regression was done for adjustments, to control for confounding and to examine the odds ratios for malaria prevalence in relation to each of the risk factors.

### Ethics

Ethical approval was sought from the University of Zambia Biomedical Research Ethics Committee (UNZABREC) and granted in February, 2013 (IRB004-11-12).

## Results

### Population characteristics

A total number of 400 participants were enrolled in this study, of these 375 had complete data. The median age of the participants was 23 years old (IQR 20-24). The median gestational stage was 24-36 weeks. Of the women enrolled 57% reported to have had a primary education while only 3.2% reported a college/university attainment. Of these women 15% had not received any SP at the time of enrollment. The major source of non-participation was refusal (<5%) and incomplete data.

### Malaria infection risk factors

The prevalence of malaria by rapid diagnostic test was 30%, by microscopy prevalence was 15% and 22% by PCR. The rapid diagnostic test is an antigen-based test and may remain positive two weeks after treatment, hence may result in false positives. Using Chi square analysis, significant results were obtained for malaria symptom knowledge and age. Over 75% of women with symptom knowledge of malaria had taken medication in the last malaria infection, with the greatest proportion (50%) having taken artemether-lumefantrine (Coartem®). In terms of insecticide-treated net distribution, 74% of the women reported to own at least one net in their household although the results suggest that there was no association with malaria. Using multivariate analysis none of the factors were significantly associated with malaria except age. Women aged 30-34 were 64% less likely to have malaria compared to those in the reference group 14-18 years old (AOR: 0.36 95% CI).

### Genotyping

**Figure 1:**
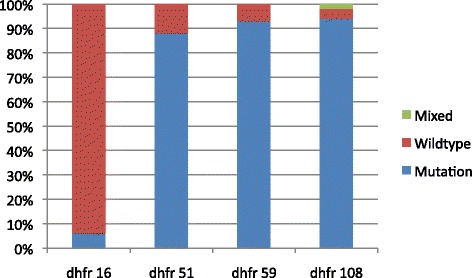
*Plasmodium falciparum* DHFR mutations (n = 72) in pregnant women of Nchelenge district, Zambia, 2013.

**Figure 2:**
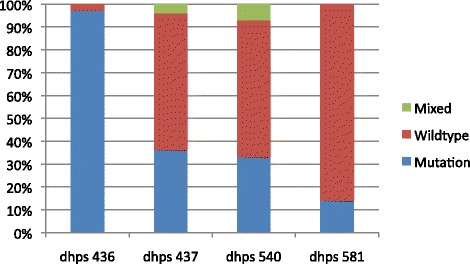
Plasmodium falciparum dhps mutations (n = 72) in Plasmodium falciparum parasites in pregnant women of Nchelenge district Zambia, 2013.

Of the PCR positives, 72 were genotyped using restriction fragment length polymorphism (RFLP) for the presence of mutations at the different loci. The prevalence of *dhfr* and *dhps* mutations are shown in Figures 1 and 2. The prevalence of the *dhfr* triple mutant (Asn-108 + Ile-51 + Arg-59) was 68% and *dhps* double mutant (Gly-437 + Glu-540) was 21%. The quintuple mutant was present in 17% (n = 12) of the samples and the sextuple mutant was present in 3% (n = 2) of the samples. The figures show additional codons at Val-16 and dhps mutation at codon 436. There was no association between SP intake and genotypes present.

## Discussion

This study documents high malaria prevalence and antifolate resistance mutations in pregnant women in a holo endemic area. It is important to note that >60% of women who were found with malaria had taken at least one dose of SP. However, with multivariate analysis there was no association between presence of malaria and any of the associated risk factors except age, hence the association found with chi square may be due to confounding. The multivariate analysis on predictors of malaria prevalence suggests an association between malaria prevalence and women aged 30-34 years compared to those women aged 14-18 years old. This may be due to a protective effect reported in other studies that older women who are likely to have had more than one previous pregnancy (multigravidae) are less likely to have malaria compared to those who have not had children previously (primigravidae) [25].

Possible bias due to non-participation may have occurred as this study was based exclusively at the health centres thereby excluding those pregnant women who did not attend antenatal clinic and who may have a different profile from those that do. Another study limitation was that clinical efficacy of SP was not determined in order to assess treatment failure, and the data collected including when the last malaria medication was taken may be subject to recall bias.

The results obtained show high levels of *dhfr* mutations indicating mostly pyrimethamine resistance but also a higher emergence of mutations Val-16 which is an indication of resistance to cycloguanil and a higher emergence of mutations at *dhps* 581 than previously reported. Another study conducted in Mansa, Zambia in pregnant women showed 63% had quintuple mutants while only two had an additional mutation at the Gly-581 loci. The treatment failure recorded in the study was 22%. This shows that the *dhps* mutations are becoming more common and there has been an escalation in the mutations in this population. This is consistent with other studies that have shown that in hyperendemic areas with high rate of transmission as is the case for Nchelenge, resistant parasites have a tendency to increase and contribute to treatment failure [26]. SP has been in use for over two decades in Zambia and due to drug pressure parasite resistance is expected to be high as is evident from trends analysis in populations were SP has been used for even shorter periods of time. Data from southern Zambia showed that by 2006, the prevalence of *dhfr* and *dhps* mutants had escalated rapidly since 1988, and that the quintuple (*dhfr* triple + *dhps* double) mutant associated with highest levels of SP clinical failure was starting to be seen (6.5%) [27].

Studies done in Tanzania have shown an emergence of the presence of the *dhps* triple mutant and suggested that IPTp with SP may not improve overall pregnancy outcome [28]. Furthermore conflicting results have been found with some studies showing increased prevalence of *dhfr* and *dhps* mutations while still others have found that SP IPTp is effective [29,30].

High prevalence of multiple mutants raises concerns as these have been shown to correlate with therapeutic or clinical failures. Therefore there is need to explore other drugs as options for IPTp.

## Conclusion

This study shows a high prevalence of malaria in pregnant women of Nchelenge district, despite reported use of SP-IPTp. This may be due to the high parasite resistance mutations to the drug as was evident in this study. The research implications are that there is need to conduct further high powered studies to determine efficacy and parasite clearance rates using SP-IPTp and also begin to explore alternative avenues for drugs to be used in IPTp if it is to be beneficial to this population.
